# Protocol to identify DNA methylation-sensitive enhancer sites

**DOI:** 10.1016/j.xpro.2026.104922

**Published:** 2026-10-29

**Authors:** Natalie Taege, Sonja C. Schriever, Henriette Kirchner

**Affiliations:** 1 Institute for Human Genetics, Division Epigenetics & Metabolism, University of Luebeck, Ratzeburger Allee 160, 23562 Luebeck, Germany; 2 Research Unit NeuroBiology of Diabetes, Institute for Diabetes and Obesity, Helmholtz Centre Munich, Ingolstädter Landstrasse 1, 85764 Neuherberg, Germany

**Keywords:** Cell Biology, Cell culture, Cell-based Assays, CRISPR, Gene Expression, Metabolism, Molecular Biology, Sequencing

## Abstract

Here, we present a protocol to identify DNA methylation-sensitive enhancer sites using the leptin-regulating enhancer RS1 in adipose tissue. We describe steps for validating RS1’s endogenous function with a dCas9-KRAB repressor system and quantifying RS1 CpG methylation by pyrosequencing. We then detail procedures for assessing methylation sensitivity by luciferase reporter assay. This protocol is adaptable to other enhancers and tissues.

For complete details on the use and execution of this protocol, please refer to Taege et al.^1^

## Highlights

• Guidance on dCas9-KRAB-mediated silencing of enhancers in differentiated adipocytes
• Steps for designing, validating, and standardizing pyrosequencing and sgRNA assays
• Steps for bisulfite pyrosequencing of CpG methylation at adipose tissue enhancers
• Instructions for cloning and *in vitro* methylation of a CpG-free luciferase reporter

## Abstract (editor highlights)

Publisher’s note: Undertaking any experimental protocol requires adherence to local institutional guidelines for laboratory safety and ethics.

## Graphical abstract

**Figure:**
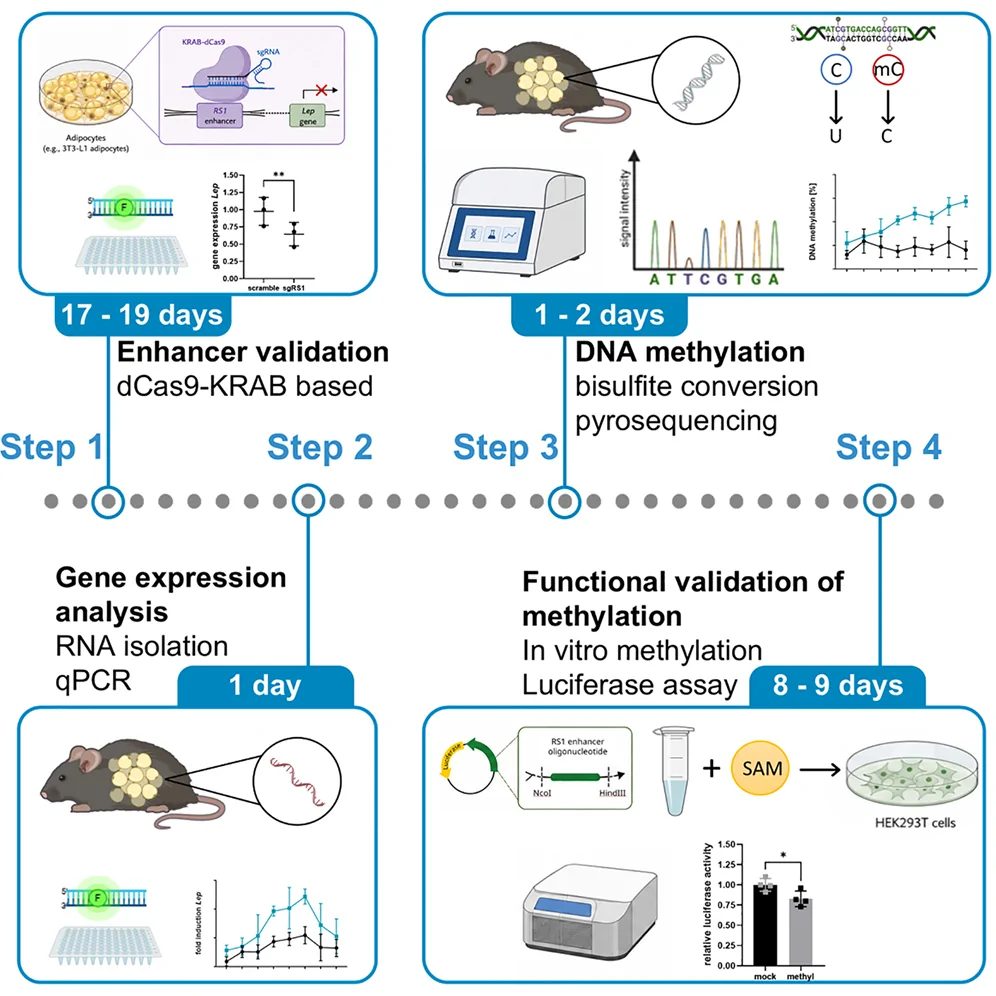

## Before you begin

This protocol describes the specific steps for identifying and analyzing DNA methylation at the *Lep* regulatory enhancer site RS1 in mouse and human adipose tissue and for functionally validating DNA methylation sensitivity using a CpG-free luciferase reporter system in HEK293T cells. The methylation analysis protocol can be adapted for other CpG sites, tissues, or species by designing appropriate PyroMark assays. The luciferase protocol can be applied to any genomic region of interest by ordering the corresponding oligonucleotide sequences. Other cell lines can be used.

### Innovation

This protocol integrates three complementary approaches to identify and validate methylation-sensitive enhancer activity. First, dCas9-KRAB-mediated epigenome editing in differentiated 3T3-L1 adipocytes enables validation of enhancer function in its native genomic and chromatin context, independently of DNA methylation manipulation. Then, quantitative bisulfite pyrosequencing links endogenous CpG methylation patterns to gene expression in primary tissue. Finally, a CpG-free luciferase reporter system (pCpGL-CMV) isolates the functional impact of insert methylation from backbone effects, ensuring that only enhancer-specific methylation is assessed. The combination of these three approaches provides multi-level evidence for methylation-sensitive enhancer activity. Normalization of pyrosequencing data to a standard curve prepared from whole genome amplified (0% methylated) and in vitro methylated (100% methylated) DNA pools enables robust comparison across multiple cohorts and experimental runs.

### Institutional permissions

All animal experiments must be performed in accordance with relevant institutional and national guidelines and regulations. Authors must obtain approval from the relevant ethics committee before conducting experiments involving animals or human subjects. Readers should acquire the necessary permissions from their own institutions before implementing this protocol.

### Preparation 1: 3T3-L1 cell culture and differentiation

**Timing: 14–16 days (perform before dCas9-KRAB transfection)**

1. Maintain 3T3-L1 preadipocytes in 3T3-L1 growth medium, materials and equipment, in a T75 flask at 37°C, 5% CO_2_.

***Note:*** Passage cells at 70–80% confluency using TrypLE for a maximum of 5 min.

2. For differentiation, seed 4 × 10^5^ cells in a 10 cm dish in 3T3-L1 growth medium. Change 3T3-L1 growth medium every other day.
3. Start differentiation protocol once cells reach 100% confluency (around 4 days post seeding):
  a. Day 0: Continue with 3T3-L1 growth medium for 1 additional day.
  b. Day 1: Change to 3T3-L1 induction medium, materials and equipment. Culture for 72 h.
  c. Day 4: Change to 3T3-L1 differentiation medium, materials and equipment.

**Figure 1:**
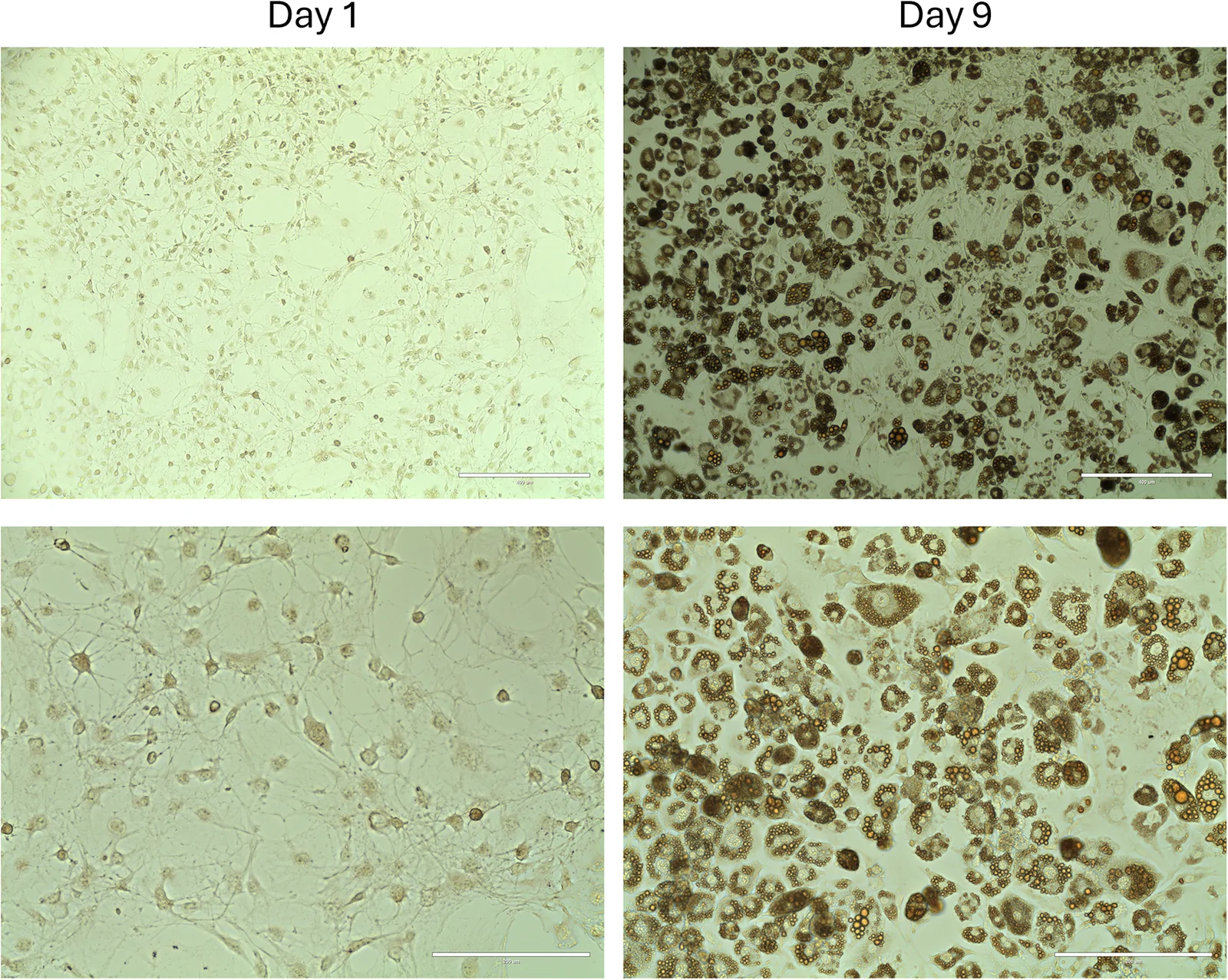
Example images of Oil-red-O staining of differentiating 3T3-L1 cultures. Cultures on day 1 and day 9 of differentiation protocol showing desired accumulation of lipid droplets and change of cell shape into round shapes can be seen. Magnification of upper row at 10x, scale bar 400 μm; lower row at 20x, scale bar 200 μm.

***Note:*** Change every 2–3 days until end of experiment (here until day 10 post differentiation, continue with step-by-step methods: dCas9-KRAB-Mediated Enhancer Silencing in Differentiated 3T3-L1 Adipocytes). Confirm successful adipocyte differentiation by visual inspection of lipid droplet accumulation using phase-contrast microscopy before proceeding to transfection. Differentiation efficiency should be >80% to ensure meaningful results (see Figure 1).

**CRITICAL:** Use antibiotic-free differentiation medium starting one day before when seeding cells for transfection to avoid antibiotic cytotoxicity.

### Preparation 2: Design of single guide (sgRNA) for target region

**Timing: 0.5 days (perform before dCas9-KRAB transfection)**

4. Retrieve the genomic sequence of the enhancer region of interest (here: *Lep* RS1) from a genome browser of your choice (e.g., UCSC Genome Browser, mm10), including ±500 bp flanking sequence to allow flexible guide placement.
5. Design 3 guide RNAs (sgRNAs, 20 nt) targeting the RS1 region using a validated sgRNA design tool such as:
  a. http://crispor.tefor.net/
  b. https://chopchop.cbu.uib.no/
  c. Benchling
6. When selecting candidate sgRNAs, apply the following criteria:
  a. Select guides with high predicted efficiency (targeting sgRNA to a specific sequence and therefore guiding the Cas enzyme to the edit site) and specificity (editing events and off-target effects).
  b. Prioritize guides with minimal predicted off-target sites, particularly those overlapping annotated promoters or exons of other genes.
  c. Ensure the 20 nt target sequence is immediately upstream of a PAM sequence (5′-NGG-3′ for SpCas9-based dCas9-KRAB).
  d. For transcriptional silencing via dCas9-KRAB, design 3 guides tiling the enhancer region rather than a single guide, as this substantially improves repression efficiency.
  e. Avoid guides targeting repetitive elements or sequences with low uniqueness such as TATA-boxes.
7. Design scramble sgRNAs (2–4) as a non-targeting control or use published sequences.
  a. The scramble sequence must have no significant complementarity to the genome of the species used (here: mouse, Mus musculus).
  b. Verify absence of off-targets using BLAST against the mouse genome (NCBI) or the design tool of your choice.
8. Order guide RNAs from a supplier of choice e.g., Trueguide sgRNAs from ThermoFisher at 1.5 nmol or Integrated DNA Technologies (IDT).

***Note:*** For optimal dCas9-KRAB-mediated repression, use 2–4 sgRNAs simultaneously targeting the enhancer region. Check the different guides separately in the sgRNA transfection step (Major Step 1, step 3) as sgRNAs can differ in their activity and specificity albeit careful design.

**CRITICAL:** Always confirm that the scramble sgRNA does not reduce *Lep* expression relative to untransfected differentiated cells by a negative transfection control (no sgRNA added) before interpreting results from RS1-targeting guides as enhancer-specific effects.

### Preparation 3: Design and validation of PyroMark assays

**Timing: 2–3 days (∼0.5 days primer design, 1–2 days annealing temperature optimization; time for primer ordering and testing material preparation excluded)**

9. Design bisulfite PCR and sequencing primers to detect methylation on CpG sites within the enhancer site(s) of interest using PyroMark Assay Design 2.0 software (Qiagen) or free web tools such as amplicondesign.^2^ When designing the primers consider the following aspects:
  a. Get the DNA sequence of the region of interest through a genome browser of your choice such as the UCSC, get the sequence of the region of interest with ±1000 additional bases as flanking region to give the design software enough sequence context and allow primer placement away from repetitive elements.
  b. Avoid placing primers over CpG sites.
    ***Note:*** Bisulfite conversion of the DNA leads to conversion of unmethylated cytosines (Cs) to uracils while methylated Cs remain, altering the sequence at CpG sites. Consequently, if primers are designed with Cs in the CpG sequence a bias towards annealing to and amplifying methylated DNA likely occurs. On the other hand, primers covering a CpG site designed with TpG amplify preferably unmethylated DNA.
  c. Using e.g., OligoAnalyzer from IDT check for
    i. CpG-rich regions (e.g., within CpG islands or the promoter region, will lead to PCR-biases)
    ii. Homopolymers (homopolymers after bisulfite conversion will lead to insufficient specificity and polymerase-slippage during sequencing)
    iii. Secondary structures
    iv. Primer dimers
    ***Note:*** As only methylated cytosines remain cytosines, bisulfite-treatment of the DNA converts the majority of the Cs in the genome to Us/Ts, leading to a DNA code which consists mostly of only the three nucleotides A, T and G. Thus, primers will show lower scores for the above listed criteria.
10. Order primers (25 nmole, HPLC-purified, standard desalted).
  a. One PCR primer per assay must carry a 5′ biotin tag for pyrosequencing immobilization.
  b. Reconstitute bisulfite PCR primers (fwd and rev) to 100 μM in 1x TE-buffer.
  c. Prepare 10 μM working stocks in 1x TE-buffer.
  d. Reconstitute sequencing primers to 100 μM in PyroMark Annealing Buffer (Qiagen).
  e. Prepare 4 μM working stocks in PyroMark Annealing Buffer.
  f. Store reconstituted primer stocks at −20°C

**Pause point:** Working stocks can be stored for 4 weeks at 4°C; avoid repeated freeze-thaw cycles.

11. Prepare 1xTAE from 50× TAE buffer, materials and equipment.
12. Optimize the annealing temperature for each bisulfite PCR assay using the PyroMark PCR Kit with DNA extracted from the same species as your samples of interest, using an identical isolation procedure and bisulfite conversion (details see Major steps 3–7):
  a. Prepare a 25 μL PCR reaction containing 12.5 μL 2x PyroMark PCR MasterMix, 2.5 μL CoralLoad, 0.2 μM each forward and reverse primer, 10–20 ng bisulfite-converted DNA.
  b. Evaluate at least three annealing temperatures or perform a gradient PCR starting at 5°C below the lowest melting temperature of the primer pair; start with 40 cycles:

**Table:** 

Steps	Temperature	Time	Cycles
Initial Denaturation	95 °C	10 min	1
Denaturation	94 °C	30 s	variable
Annealing	variable	25 s	
Extension	72 °C	25 s	
Final extension	72 °C	10 min	1
Hold	4 °C	forever	–

13. Run PCR products on a 1% agarose gel in 1xTAE with SYBR Safe DNA gel stain and 100 bp DNA ladder. Image the gel on the ChemiDoc Touch or an equivalent imaging system.

**Figure 2:**
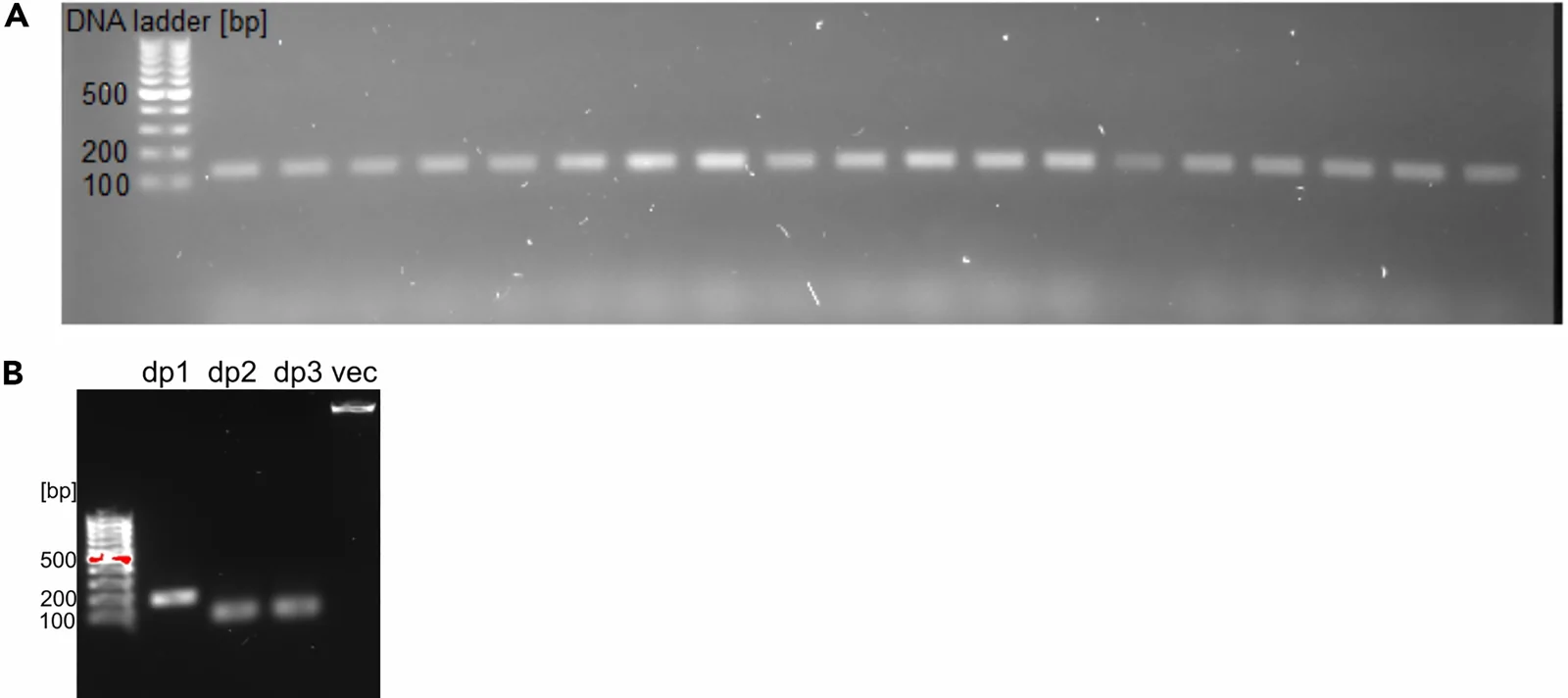
Representative images of 1% agarose gel. Gel stained with SYBR Safe of (A) the RS1 Lep enhancer PCR product after bisPCR showing strong, single bands and of (B) example inserts (dp1 – dp3) and vector (vec) for gel purification.

***Note:*** Select the annealing temperature yielding the strongest, cleanest band. If bands appear weak but clean, increase the cycles to up to 45 (see Figure 2 A as example for a clean, strong band).

14. Validate each assay by pyrosequencing for primer interactions and self-priming artifacts using the following control combinations with pyrosequencing (volumes are 10 μL + 2 μL):
  a. Biotinylated primer + annealing buffer
  b. Biotinylated primer + PCR product
  c. PCR product + annealing buffer
  d. Sequencing primer + biotinylated primer.

**Figure 3:**
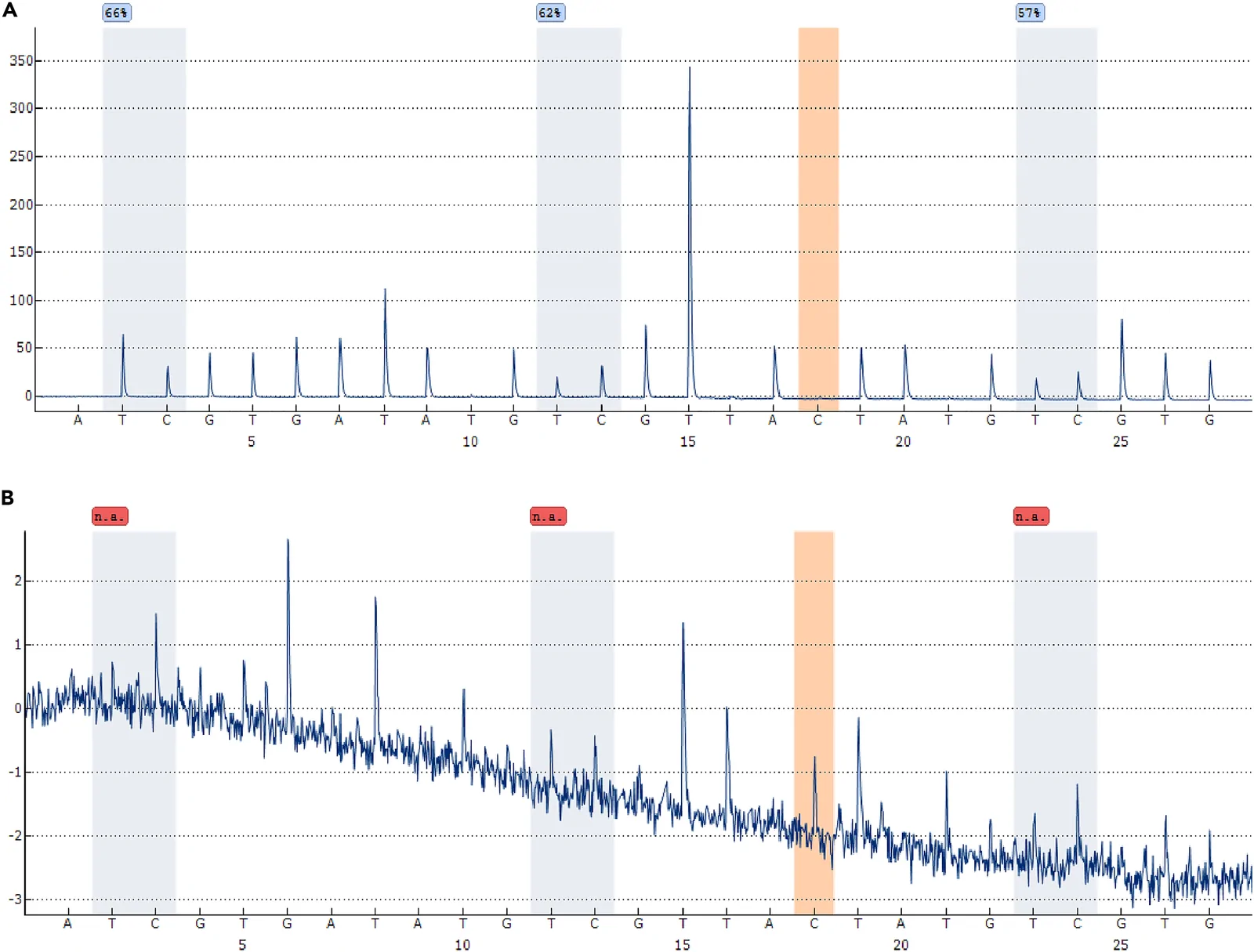
Representative pyrosequencing outputs (pyrogram) by the pyrosequencer. Pyrograms are analyzed automatically by the PyroMark Q48 Autoprep software (A) Pyrogram for the RS1 assay (biotinylated primer + PCR product). (B) Pyrogram of quality control (biotinylated primer + sequencing primer) showing unspecific peaks with low peak height, as expected for a properly designed assay. The sequence to analyze for the specific assay, the percentages methylation at each CpG position as well as an orange box indicating an internal control included automatically by the software and machine to control for incomplete bisulfite conversion are shown. Grey boxes indicate the histogram of the expected peak heights.

***Note:*** Make sure not to load annealing primer into the cartridge of the PyroMark Q48 Autoprep. Exclude any assay showing a detectable pyro-signal above the peak height of 15 in these controls. An example for a derived pyrogram of these controls can be found in Figure 3. For the mouse and human *LEP* RS1 assays, the optimized annealing conditions are 49.5°C, 40 cycles (mouse) and 52.9°C, 45 cycles (human), respectively.

### Preparation 4: Preparation of standard curve DNA pools (pyrosequencing)

**Timing: 2–3 days (perform once; can be used for multiple assays)**

Standard curves are required to correct for assay-specific PCR and sequencing biases, including primer insufficiencies that can cause non-linear signal generation depending on the proportion of methylated DNA present. Normalization to the standard curve accounts for these non-linearities, corrects for run-to-run variation, and ensures accurate, comparable methylation values across independent experimental runs.

15. Prepare 0% methylated DNA by whole-genome amplification (WGA) of genomic mouse DNA using the REPLI-g Mini Kit (this DNA can be used universally across all murine assays):
  a. Denature DNA (in our case 400 ng total mouse DNA of epididymal adipose tissue, V = 2.5 μL) by adding 2.5 μL Buffer D1 and incubating for 3 min at 22 ± 1°C.
  b. Add 5 μL N1 buffer, centrifuge briefly.
  c. Add a mix of 10 μL DEPC-treated H2O, 29 μL REPLI-g Mini Reaction Buffer, and 1 μL REPLI-g Mini DNA Polymerase.
  d. Incubate at 30°C for 16 h.
  e. Inactivate the polymerase at 65°C for 3 min.
  f. Purify WGA product by drop dialysis:
    i. Pipette product on a 0.1 μm nitrocellulose membrane floating on Milli-Q water for ≥5 min.^3^
    ii. Recover DNA droplet, quantify, and store at −20°C.
16. Prepare 100% methylated DNA by in vitro methylation of genomic DNA with M.SssI CpG methyltransferase (this DNA can be used universally across all murine assays):
  a. Dilute S-adenosylmethionine (SAM) stock (32 mM) 1:20 in nuclease-free water to obtain 1600 μM working solution.
  b. Set up the in-vitro methylation reaction (In vitro methylation reaction, materials and equipment) by adding 2 μL of 10x NEB buffer 2, 1 μL SAM as donor for the methyl group, plasmid DNA, 1 μL M.SssI methyltransferase and nuclease-free water to a final reaction volume of 20 μL.
  c. Incubate at 37°C for 4 h, then heat-inactivate at 65°C for 20 min.
  d. Purify by drop dialysis as described above in 15f and store at −20°C.
17. Mix 0%- and 100%-methylated DNA to prepare standards at 0%, 25%, 50%, 75%, and 100% methylation.
18. Bisulfite-convert all standard DNA pools (see Major step 2: Bisulfite Conversion of Genomic DNA) and pyrosequence each standard with the relevant assay to generate the standard curve.

**Pause point:** Standard curve DNA pools can be stored at −20°C for up to 1 year.

***Note:*** For human samples, ready-made 0% and 100% bisulfite controls are commercially available (EpiTect PCR Control DNA Set, Qiagen).

### Preparation 5: Cloning of the RS1 insert into pCpGL-CMV (luciferase assay)

**Timing: 1 week (perform once)**

19. Order the RS1 insert as complementary single-stranded oligonucleotides with NcoI (5′-C/CATGG-3′) and HindIII (5′-A/AGCTT-3′) overhangs.
  ***Note:*** If the insert cannot be ordered as a custom single-stranded oligonucleotide (e.g., due to length constraints, typically >200 bp), the insert can be generated by PCR and cloned using restriction digestion and ligation as described below.
  a. Design primers containing the insert sequence of interest flanked by overhangs carrying the recognition sequence and include at least 3 bp of additional sequence beyond the recognition site to allow efficient enzyme binding and cutting.
  b. Check the full insert sequence for internal occurrences of the chosen restriction enzyme recognition sites. If the recognition sequence is present within the insert, select alternative restriction enzymes that do not cut internally.
  c. Verify that the two chosen restriction enzymes are compatible in the same buffer (here: rCutSmart Buffer supports both NcoI-HF and HindIII-HF) to allow simultaneous double digestion.
  d. Consider the final insert-to-vector size ratio; very small inserts (<50 bp) ligate inefficiently and may require adjusted molar ratios.
20. Anneal the oligonucleotides: mix equimolar amounts in annealing buffer, heat to 95°C for 5 min, then cool with gradual temperature decrease (−1 °C/min) to 22 ± 1°C.
21. Set up separate digestion reactions for vector (500 ng pCpGL-CMV) and insert on ice:

**Table:** 

Reagent	Vector (1×)/Insert of PCR product
NcoI-HF (20 units/μL)	1 μL
HindIII-HF (20 units/μL)	1 μL
rCutSmart Buffer (10×)	5 μL
DNA (plasmid ∼500 ng or PCR product ∼0.5–1 μg)	variable
Nuclease-free H2O	to 50 μL
**Total**	**50 μL**

22. Incubate at 37°C for 15 min.
23. Dephosphorylate vector:
  a. Add 1 μL Quick CIP per ∼1 μg DNA (approximately 1 pmol ends for a 3 kb plasmid).
  b. Incubate at 37°C for 10 min.

**CRITICAL:** Only perform this step if PCR was used to generate and amplify the insert, not necessary if oligonucleotides were ordered. Dephosphorylation is performed on the vector only. Dephosphorylating the insert will prevent ligation.

24. Heat-inactivate all reactions at 80°C for 20 min.
25. Run all digestion products on a 1% agarose gel in 1× TAE buffer with gel stain of choice (e.g., SYBR Safe DNA gel stain, example shown in Figure 2 B).
26. Excise bands of the expected size under appropriate illumination depending on gel stain of choice (here UV or 470–530nm) using a clean scalpel.

**CRITICAL:** If using UV-light, minimize UV exposure time to reduce DNA damage.

27. Purify DNA from gel slices using a gel extraction kit (e.g., WIZARD SV Gel and PCR Clean-up system) according to the manufacturer’s instructions.
28. Quantify purified vector and insert using the Qubit dsDNA BR Assay.
29. Ligate the duplexed insert upstream of the CMV promoter and luciferase reporter using Quick Ligase in a 50 μL reaction containing 1x Quick ligase reaction buffer and a molar ration of 1:3 vector to insert, nuclease-free water and 1 μL Quick ligase for 10 min at 22 ± 1°C.
30. Transform into PIR1 OneShot® E.coli (essential for pCpGL-CMV vector amplification), select a minimum of 3 colonies, and sequence to verify correctness of inserts.

***Note:*** Verify correct insertion by Sanger sequencing or other sequencing methods such as long-read sequencing, services available from various companies, using a vector-specific primer flanking the cloning site before proceeding.

31. Purify verified plasmid using an endotoxin-free Mini or Midiprep kit and store at −20°C.

***Note:*** pCpGL-CMV was a gift from Prof. Rehli.^4^ Requests for this plasmid should be directed to the original provider or via Addgene.

## Key resources table

**Table:** 

REAGENT or RESOURCE	SOURCE	IDENTIFIER
**Biological samples**		

3T3-L1 preadipocyte cell line	ATCC	RRID:CVCL_0123
HEK293T cells	ATCC	RRID:CVCL_0063
Human adipose tissue (60–100 mg)		N/A
Mouse adipose tissue (60–100 mg)	C57BL/6	N/A

**Chemicals, peptides, and recombinant proteins**		

20X TE	Promega	Cat#A2651
3-Isobutyl-1-methylxanthine (IBMX)	Sigma-Aldrich	Cat#I-7018
Agarose Broad Range	Carl Roth	Cat# 9012–36–6
Dexamethasone	Sigma-Aldrich	D4902
Dulbecco’s Modified Eagle Medium (DMEM), high glucose	GIBCO, Life Technologies	Cat#31966021
Fetal Bovine Serum One Shot	GIBCO, Life Technologies	Cat#A5209402
GeneRuler 100 bp DNA Ladder	Thermo Fisher Scientific	Cat#SM0243
HindIII-HF	New England Biolabs	Cat#R3104
Insulin (e.g., Humalog)	Eli Lilly	VL7510
Lipofectamine 2000	Thermo Fisher Scientific	Cat#11668027
M.SssI CpG Methyltransferase	New England Biolabs	Cat#M0226L
NcoI-HF	New England Biolabs	Cat#R3193
Opti-MEM I Reduced Serum Medium	Thermo Fisher Scientific	Cat#31985062
Penicillin-Streptomycin	GIBCO, Life Technologies	Cat#15070063
Quick Ligase	New England Biolabs	Cat#M2200
rCutSmart Buffer (10x)	New England Biolabs	Cat#B6004S
S-adenosylmethionine (SAM), 32 mM	New England Biolabs	Supplied with M0226L
SYBR Safe DNA Gel Stain	Thermo Fisher Scientific	Cat#S33102
Tryp-LE	GIBCO, Life Technologies	Cat#25200056

**Critical commercial assays**		

3T3-L1 Cell Avalanche® Transfection Reagent	EZ Biosystems	Cat#EZT-3T3L1-1
Dual-Glo Luciferase Assay System	Promega	Cat#E2920
EpiTect Fast DNA Bisulfite Kit	Qiagen	Cat#59824
High-Capacity cDNA Reverse Transcription Kit	Thermo Fisher Scientific	Cat#4374966
miRNeasy Mini Kit or RNeasy Mini Kit	Qiagen	Cat#217004 Cat#74104
PrimeTime Gene Expression Master Mix	IDT	Cat#1055771
PyroMark PCR Kit	Qiagen	Cat#978703
PyroMark Q48 Advanced CpG Kit	Qiagen	Cat#974022
QIAamp Fast DNA Tissue Kit	Qiagen	Cat#51404
QIAamp Mini Kit	Qiagen	Cat#51304
Qubit dsDNA BR Assay Kit	Thermo Fisher Scientific	Cat#Q32850
REPLI-g Mini Kit (WGA)	Qiagen	Cat#150023

**Oligonucleotides**		

PrimeTime assay *Hprt*	Integrated DNA Technologies, US	Mm.PT.39a.22214828
PrimeTime assay *Lep*	Integrated DNA Technologies, US	Mm.PT.58.13515402
PrimeTime assay *LEP*	Integrated DNA Technologies, US	Hs00174877_m1
PrimeTime assay *RPL37A*	Integrated DNA Technologies, US	Hs01102345_m1
BisPCR forward primer, mouse RS1	Integrated DNA Technologies, US	GGGTAGTTGAAATAATTAGTTAGT
BisPCR reverse primer, mouse RS1 (biotinylated reverse primer for pyrosequencing)	Integrated DNA Technologies, US	/bio-CCTCAAAACTACTCCAAACATACA
Pyrosequencing primer, mouse RS1	Integrated DNA Technologies, US	AATTGGTGGTTTTGGTATTA
BisPCR forward primer, human RS1	Integrated DNA Technologies, US	GTAGTAGGGGTATTATTATAGGATTAT ATTGA
BisPCR reverse primer, human RS1 (biotinylated reverse primer for pyrosequencing)	Integrated DNA Technologies, US	/bio-ACAAAATATTCTTCC AATCAATTCTTTAAC
Pyrosequencing primer, human RS1	Integrated DNA Technologies, US	TTGAAGAAGTATTGGTTAAGAAG
RS1 insert oligonucleotides (sense + antisense, for pCpGL-CMV cloning)	Integrated DNA Technologies, US	AGCTTTCTGGCATCATCGTGACCA GCGGTTTTTCCCACAGCGG TGTCCTAACAC CATGGTGTTAGGACACCGC TGTGGGAAAAACCGCTGGTCACG ATGATGCCAGAA
sgRNAs targeting Lep RS1 (mouse)	This study, ordered from ThermoFisher Scientific	1. ACCGCUGUGGGAAAAACCGC 2. GCAUGUUAGGACACCGCUGU 3. AGCAUGUUAGGACACCGCUG
Scramble sgRNA (non-targeting control)	This study, ordered from ThermoFisher Scientific, sequence from Suzuki et al.^5^	GCUUAGUUACGCGUGGACGA

**Recombinant DNA**		

pCpGL-CMV (CpG-free firefly luciferase vector)	Klug et al.^4^	N/A
pLX303-Zim3-KRAB-dCas9	Addgene	#154472
pRL-SV40 Renilla-SV40 (internal transfection control)	Promega, US	#E2231

**Software and algorithms**		

Image Lab (ChemiDoc analysis) version 6.01.34	Bio-Rad Laboratories GmbH	RRID:SCR_014210
Microsoft Excel (Office 15 & 16)	Microsoft	RRID:SCR_016137
PyroMark Assay Design 2.0 (version 2.0.2.5, license needed)	Qiagen	https://www.qiagen.com/de-us/resources/operatingsoftware/pyromark-assay-design-software-v-2-0
PyroMark Q48 Autoprep software (version 2.4.2, license needed)	Qiagen	https://www.qiagen.com/de-us/resources/operatingsoftware/sow-686-0-02-pyromark-q48-autoprep-application-software
QuantStudio Design and Analysis software (version 2.8.0.0)	ThermoFisher Scientific	https://www.thermofisher.com/de/de/home/technical-resources/software-downloads/quantstudio-3-5-real-time-pcr-systems.html

**Other**		

10 cm dish	e.g., Sarstedt	Cat#73.3900
12-well cell culture plate	e.g., Sarstedt	Cat#83.3921
96-well white plates (luciferase reading)	e.g., Sarstedt	N/A
ChemiDoc Touch Imaging System	Bio-Rad	RRID:SCR_019037
Fisherbrand Bead Mill 24 Homogenizer	Fisher Scientific,	Cat# 15515799
Incubator with CO2 CellXpert	e.g., Eppendorf SE	N/A
Neubauer counting chamber	e.g., VWR	N/A
Nitrocellulose membrane filters 0.1 μm VCWP	Merck Millipore, Ireland	Cat# VCWP02500
PyroMark Q48 Autoprep	Qiagen	N/A
QuantStudio 5 Real-Time PCR System	Thermo Fisher Scientific	RRID:SCR_020240
Qubit 4 Fluorometer	Thermo Fisher Scientific	RRID:SCR_026883
T75 cell culture flask	e.g., Sarstedt	Cat#83.3911.002
Thermal cycler (e.g., Mastercycler nexus gradient)	e.g., eppendorf	Cat# 6331000041

## Materials and equipment

**Table:** 3T3-L1 Growth Medium

Reagent	Volume
DMEM, high glucose (4.5 g/L)	445 mL
FBS	50 mL (10% v/v)
Penicillin-Streptomycin (100x)	5 mL (1x final)

Store at 4°C. Warm to 37°C before use.

**Table:** 3T3-L1 Induction Medium (differentiation initiation, 72 h)

Reagent	Amount
3T3-L1 growth medium (as above)	Base
Insulin (3.5 mg/mL stock)	1 μg/mL
Dexamethasone (50 μM work stock in water)	0.25 μM
IBMX (200 mM work stock in DMSO)	0.5 mM

Prepare fresh.

**Table:** 3T3-L1 Differentiation Medium (used from day 3 on until end of experiment)

Reagent	Amount
3T3-L1 growth medium (as above)	Base
Insulin (3.5 mg/mL stock)	1 μg/mL

Prepare fresh. Change every 2–3 days.

**Table:** dCas9-KRAB Transfection Mix (per well, 12-well plate)

Reagent	Amount per well
dCas9-KRAB plasmid	300 ng
Opti-MEM	12 μL
3T3-L1 Cell Avalanche® Transfection Reagent	0.4 μL

Prepare fresh.

**Table:** sgRNA Transfection Mix (per well, added 4.5 h after plasmid transfection)

Reagent	Amount per well
sgRNA	15 pmol
Opti-MEM	50 μL
3T3-L1 Cell Avalanche® Transfection Reagent	0.4 μL

Prepare fresh on ice.

**Table:** cDNA Synthesis Reaction Mix (per 20 μL reaction, High-Capacity RT Kit)

Reagent	Volume per sample
10× RT Buffer	2 μL
25× dNTP Mix (100 mM)	0.8 μL
10× RT Random Primers	2 μL
MultiScribe™ Reverse Transcriptase	1 μL
RNase Inhibitor	1 μL
Nuclease-free H2O	3.2 μL
RNA sample (up to 1 μg)	10 μL

Prepare fresh on ice.

**Table:** qPCR Reaction Mix (per 10 μL reaction)

Reagent	Volume per sample
PrimeTime Master Mix	5 μL
PrimeTime qPCR assay 1 (20×, target gene: *Lep*)	0.5 μL
PrimeTime qPCR assay 2 (20×, housekeeper: *Hprt*) or DEPC-treated H2O (singleplex)	0.5 μL
cDNA (5 ng/μL)	4 μL

Multiplex Lep and Hprt in the same well or run as separate singleplex reactions depending on assay compatibility. Use the same master mix type consistently across all samples within one experiment. Use the appropriate assays/primers for your organism of choice. Prepare fresh on ice.

**Table:** 50× TAE Buffer (1 L)

Reagent	Amount
Tris base	242 g
Glacial acetic acid	57.1 mL
0.5 M EDTA (pH 8.0)	100 mL
ddH2O	to 1 L

Store at 22 ± 1°C for up to a year. Dilute to 1× with ddH2O before use.

**Table:** Tissue Lysis Buffer (QIAamp Fast DNA Tissue Kit)

Reagent	Volume per sample
Buffer AVE	200 μL
Buffer VXL	40 μL
DX Reagent	1 μL
Proteinase K	20 μL
RNase A	4 μL

Prepare fresh immediately before use.

**Table:** Bisulfite Conversion Reagent Volumes (EpiTect Fast DNA Bisulfite Kit)

Input DNA volume	DNA protect Buffer	Bisulfite solution
≤20 μL (≥1,000 ng)	35 μL	85 μL
40 μL (<1,000 ng)	15 μL	85 μL

When input DNA is <100 ng, add Carrier-RNA to Buffer BL as per manufacturer’s instructions.

**Table:** BisPCR Master Mix (per 25 μL reaction)

Reagent	Volume (1 reaction)	Final concentration
PyroMark PCR MasterMix (2×)	12.5 μL	1×
CoralLoad Concentrate (10×)	2.5 μL	1×
Forward primer (10 μM)	0.5 μL	0.2 μM
Reverse primer (10 μM)	0.5 μL	0.2 μM
Bisulfite-converted DNA	1 μL	10–20 ng
RNase-free water	8 μL	—
Total	25 μL	—

Always prepare master mix fresh on ice. Do not vortex primers if ice crystals are still present. Avoid multiple freeze–thaw cycles of primers.

**Table:** In Vitro Methylation Reaction (20 μL total)

Reagent	Volume
10× NEB Buffer 2	2 μL
SAM (1600 μM, freshly diluted from 32 mM stock 1:20)	1 μL
Plasmid DNA (target: 750–1000 ng)	Variable (max. 25% of total volume)
M.SssI	1 μL
Nuclease-free water	to 20 μL

Mock reaction: identical setup but omit M.SssI; replace with additional nuclease-free water. Prepare fresh on ice.

**Table:** HEK293T growth medium

Reagent	Volume
DMEM, high glucose (4.5 g/L)	445 mL
FBS	50 mL (10% v/v)
Penicillin-Streptomycin (100x)	5 mL (1x final)

Store at 4°C. Warm to 37°C before use.

**Table:** Transfection Mix (3 wells per condition in 96-well plate)

Reagent	Volume (per well)	Volume (*N* = 3 wells + excess)
Lipofectamine 2000	0.2 μL	0.8 μL
Opti-MEM (Lipofectamine step)	4.8 μL	19.2 μL
Plasmid (methylated or mock, 50 ng/μL)	2 μL	8 μL
Renilla-SV40 (1 ng/μL)	1 μL	4 μL
Opti-MEM (DNA step)	2 μL	8 μL
Total transfection volume added per well	10 μL	—

Cells are seeded in 100 μL antibiotic-free growth medium per well; total volume after transfection addition: 110 μL. Prepare fresh at 22°C ± 1°C.

## Step-by-step method details

### dCas9-KRAB-mediated enhancer silencing in differentiated 3T3-L1 adipocytes

**Timing: 3 days (performance of preparation 1 strictly necessary)**

This step uses a catalytically inactive Cas9 fused to the KRAB transcriptional repressor domain (dCas9-KRAB) to silence the RS1 enhancer in fully differentiated 3T3-L1 adipocytes. This approach interrogates enhancer function in its native genomic context. *Lep* expression following dCas9-KRAB targeting is compared to cells transfected with a non-targeting scramble sgRNA to determine whether RS1 contributes to Lep transcriptional regulation independently of DNA methylation.

1. Day 1: Cell Seeding
  a. After 10 days of differentiation in the 10 cm dish (see Preparation 1), transfer 3T3-L1 adipocytes to a 12-well plate:
    i. Wash cells with 1× DPBS, trypsinize, add antibiotic-free differentiation medium, gently pipette up and down
    ii. Count and seed cells at 4∗10^4^ cells/well on a 12 well plate (one 10 cm dish is sufficient for 1.5–2 12 well plates).
    iii. Allow cells to recover for 24 h at 37°C, 5% CO_2_ before transfection.
2. Day 2: Transfection
  a. Plasmid transfection (dCas9-KRAB): Prepare transfection mix per well as described in dCas9-KRAB transfection mix, materials and equipment. Vigorously mix all components by pipetting or vortexing, incubate for 15 min at 22 ± 1°C, and add dropwise to the well.
  b. Centrifuge the plate at 300 × g for 5 min.
  c. Incubate at 37°C, 5% CO_2_ for 4.5 h, then change to fresh antibiotic-free differentiation medium.
  d. sgRNA transfection: Prepare sgRNA transfection mix per well as described in sgRNA transfection mix, materials and equipment. Vigorously mix by pipetting or vortexing, incubate for 15 min at 22 ± 1°C, and add dropwise to each well.
  e. Centrifuge at 300 × g for 5 min.
  f. After 4.5 h, change to fresh 3T3-L1 differentiation medium.
  g. Incubate for 36 h at 37°C, 5% CO_2_.
3. Day 3: Harvest
  a. Harvest cells for downstream RNA extraction by washing the cells once with cold 1× DPBS and adding Qiazol for lysis. Continue with RNA isolation and qRT-PCR to quantify *Lep* expression (see next step).

**CRITICAL:** Always include a scramble sgRNA (non-targeting, no complementarity to the mouse genome) as a transfection control in parallel to the RS1-targeting sgRNA. This controls for non-specific effects of the transfection procedure and dCas9-KRAB overexpression on *Lep* expression. Transfect 3 different sgRNAs separately as sgRNAs can differ in their activity and specificity. When testing sgRNAs and specifically the scramble RNA, always include a negative transfection control (no sgRNA added) to check for off-target effects of the scramble RNA on gene expression read-out of interest.

***Note:*** Sequential transfection of plasmid and sgRNA (separated by 4.5 h) is necessary because 3T3-L1 adipocytes are difficult to transfect and benefit from the reduced toxicity of a two-step approach. Choose the appropriate cell line reflecting your tissue of interest.

**Pause point:** Cells can be lysed and stored as RNA at −80°C after harvest before proceeding to qRT-PCR.

### RNA extraction, cDNA synthesis, and quantitative RT-PCR quantification of *Lep* expression

**Timing: 1 day**

This step quantifies the effect of dCas9-KRAB-mediated RS1 silencing on *Lep* mRNA expression in differentiated 3T3-L1 adipocytes and can be applied to check gene expression levels of interest. The housekeeping gene *Hprt* is used for normalization.

4. RNA Extraction
  a. Extract total RNA using the miRNeasy Mini Kit (used here) or RNeasy Mini Kit (Qiagen) according to the manufacturer’s instructions using Qiazol. Include a on-column DNase digestion step as recommended and instructed by the manufacturer.
  b. Elute RNA in RNase-free water and quantify using NanoDrop or equivalent.

**Pause point:** RNA can be stored at −80°C until cDNA synthesis.

5. cDNA Synthesis.
  a. Dilute RNA to the desired concentration in nuclease-free water (typically targeting a total of 500 ng or 1000 ng (50 ng/μL or 100 ng/μL). Prepare the cDNA synthesis master mix on ice as described in cDNA synthesis reaction mix, materials and equipment.
  b. Add 10 μL RNA (50 ng/μL or 100 ng/μL) per reaction tube, mix gently, and centrifuge briefly.

**Table:** 

Steps	Temperature	Time
Primer annealing	25°C	10 min
Reverse transcription	37°C	120 min
Enzyme inactivation	85°C	5 min
Hold	4°C	forever
  c. Run the following thermal cycling program:
  d. Dilute cDNA in nuclease-free water to a working concentration of 5 ng/μL.
    ***Note:*** Always include a no-template control (NTC; water instead of RNA) and a no-reverse-transcriptase control (no-RT; omit MultiScribe™ reverse transcriptase) per RNA batch to detect contamination and genomic DNA carryover, respectively.
    **Pause point:** cDNA can be stored at −20°C.
6. qPCR.
  a. Prepare the qPCR reaction mix on ice (qPCR Reaction Mix, materials and equipment) as master mix.
  b. Pipette 6 μL master mix into each well of a 96-well qPCR plate.
  c. Add 4 μL diluted cDNA (5 ng/μL) per well. Seal the plate with optical adhesive film, carefully vortex and centrifuge briefly at 500 × *g*.
  d. Run the plate on the QuantStudio 5 Real-Time PCR System using the standard cycling program appropriate for the master mix used.
  e. Analyze results using the ThermoFisher Design and Analysis software. Export Ct values for *Lep* (approx. 29) and *Hprt* (approx. 24) per sample.

**CRITICAL:** Use the same Ct threshold for *Lep* and *Hprt* across all samples being directly compared. Changing thresholds between runs introduces systematic errors that cannot be corrected post hoc.

***Note:*** Run each sample in technical duplicates. Exclude technical replicates with a Ct standard deviation > 0.5 from analysis.

### DNA isolation from adipose tissue

**Timing: 1–3 h**

This step produces genomic DNA from frozen adipose tissue of sufficient purity and quantity for bisulfite conversion and subsequent methylation analysis. The QIAamp Fast DNA Tissue Kit (preferred for large tissue amounts) or the QIAamp Mini Kit can be used interchangeably.

7. Keep adipose tissue on dry ice until transferred.
8. Weigh 60–100 mg adipose tissue on dry ice.
9. Transfer tissue to a 2 mL screw-cap micro tube containing 3–4 cell lysis beads.
10. Prepare tissue lysis buffer, materials and equipment, and add to the tube.
11. Homogenize using a bead mill (2 cycles; pause 5 s between cycles; homogenization time 15 s per cycle at the instrument’s high-speed setting).

***Alternatives:*** For the disruption of cells, other mechanical alternatives to cell lysis beads and bead mill could be used such as tissue homogenizers.

12. Proceed with the QIAamp Fast DNA Tissue Kit or QIAamp DNA Mini Kit according to the manufacturer’s instructions.
13. Elute DNA in 50 μL DEPC-treated H2O. The expected yield ranges from 70–200 ng/μL.

**Pause point:** Isolated DNA can be stored at −20°C for several months or at −80°C for long-term storage before proceeding.

***Alternatives:*** Other DNA extraction kits including DNA purification steps might work equally well (such as DNA from AllPrep DNA/RNA/Protein Mini Kit, Qiagen).

### DNA quantification

**Timing: 30 min**

Accurate DNA quantification is required to use the correct input amount for bisulfite conversion.

14. Prepare Qubit dsDNA BR working solution by diluting Qubit dsDNA BR Reagent 1:200 in Qubit dsDNA BR Buffer.
15. Calibrate the Qubit 4 fluorometer on each measurement day:
  a. Prepare 2 calibration tubes by mixing 190 μL working solution with 10 μL of Standard 1 (S1) and Standard 2 (S2), respectively.
  b. Insert into the Qubit 4 and run the calibration routine.
16. Prepare sample measurement tubes: add 199 μL working solution to Qubit 0.5 mL PCR tubes.
17. Add 1 μL of each DNA sample to its tube, mix by pipetting, and incubate at 22 ± 1°C for at least 2 min in the dark.
18. Measure on the Qubit 4 using the built-in dsDNA BR protocol; the instrument automatically reports the concentration in the original sample tube using the input volume that was set when starting the Qubit assay.

***Note:*** The Qubit dsDNA BR Assay is preferable to absorbance-based methods (e.g. NanoDrop) because it specifically detects double-stranded DNA and is insensitive to RNA, single-stranded DNA, and contamination (e.g. protein, phenol, salts).

### Bisulfite conversion of genomic DNA

**Timing: ∼2.5 h (hands-on: ∼30 min)**

Bisulfite conversion chemically deaminates unmethylated cytosines to uracil while methylated cytosines remain unchanged, enabling discrimination of methylation status at single-CpG resolution.

19. Use 1000 ng DNA where possible. Adjust volume and buffer, especially the elution buffer, accordingly (Bisulfite conversion reagent volumes, materials and equipment).
20. Set up the bisulfite conversion reaction in a PCR tube according to the EpiTect Fast DNA Bisulfite Kit, adding DNA, bisulfite solution, and DNA Protect Buffer.
21. Run the following thermal cycling program:

**Table:** 

Steps	Temperature	Time
Denaturation	95°C	5 min
Incubation	60°C	30 min
Denaturation	95°C	5 min
Incubation	60°C	20 min
Hold	20°C	Until use

22. Perform clean-up and desulfonation steps as instructed by the manufacturer, including all recommended drying and washing steps.
23. Elute bisulfite-converted DNA in 15–50 μL Buffer EB, targeting ∼20 ng/μL relative to input DNA. Record elution volume.

**CRITICAL:** Include all drying and washing steps during clean-up; incomplete desulfonation reduces PCR amplification efficiency.

***Note:*** For input DNA < 100 ng, add Carrier-RNA to Buffer BL as specified by the manufacturer to prevent adsorption of low-concentration DNA to the membrane.

**Pause point:** Bisulfite-converted DNA is stable at −20°C for several weeks. Avoid repeated freeze–thaw cycles as this can degrade the converted DNA.

### Bisulfite PCR

**Timing: ∼3 h (including gel electrophoresis)**

BisPCR amplifies the region of interest from bisulfite-converted DNA (bisDNA) using assay-specific primers. During amplification, bisulfite-converted uracils are converted to thymines. One primer carries a 5′ biotin tag required for pyrosequencing.

24. Thaw bisulfite-converted DNA, primers, and PyroMark PCR MasterMix on ice.
25. Prepare master mix on ice as described in materials and equipment. Prepare one additional H_2_O (no-template) control reaction.
26. Pipette 24 μL master mix (without DNA) into DNA-free PCR strip tubes.
27. Add 1 μL bisulfite-converted DNA to each tube. This amount can be adjusted depending on bisDNA yield. Pipette up and down carefully to ensure complete transfer.
28. Mix by brief gentle vortexing and centrifuge.
29. Run the following PyroMark cycling program:

**Table:** 

Steps	Temperature	Time	Cycles
Initial denaturation	95°C	10 min	1
Denaturation	94°C	30 s	40 (mouse)/45 (human)
Annealing	49.5°C (ms)/52.9°C (hu)	25 s	
Extension	72°C	25 s	
Final extension	72°C	10 min	1
Hold	4°C	max. 16 hours

***Note:*** The annealing temperature is assay specific. For other regions, optimize as described in Preparation 1.

**Pause point:** PCR products can be stored at −20°C for longer storage.

30. Verify the PCR product by agarose gel electrophoresis:
  a. Prepare a 1% agarose gel in 1× TAE buffer with 1 μL SYBR Safe DNA Gel Stain per 10 mL gel.
  b. Load 4 μL of PCR product alongside GeneRuler 100 bp DNA Ladder.
  c. Run at 150 V for 20–25 min (times and voltage depends on used electrophoresis chamber size) in 1× TAE buffer.
  d. Image the gel on the ChemiDoc Touch or an equivalent imaging system and analyze band intensity using Image Lab software or equivalent (example shown in Figure 2A).

**CRITICAL:** Only proceed to pyrosequencing if a single, specific band of the expected size is visible and the no-template control is negative.

### Pyrosequencing

**Timing: ∼2 h (after PCR product verification)**

CpG pyrosequencing on the PyroMark Q48 Autoprep provides nucleotide-resolution quantification of DNA methylation at each CpG site within the sequenced region.

31. Program the PyroMark Q48 Autoprep software with the CpG sequencing dispensation order for the relevant assay.
32. Load and prepare the PyroMark Q48 Autoprep cartridge and disc according to the manufacturer’s instructions, using the PyroMark Q48 Advanced CpG Kit. Use the assay-specific sequencing primer (4 μM in annealing buffer) prepared in Preparation 3.
33. Load each well of the disc with 3 μL streptavidin-coated magnetic beads and 10 μL BisPCR product (15 μL for assays requiring higher input, e.g., sorted adipocyte samples).
34. Start the run; the instrument automatically performs bead capture, washing, denaturation, annealing, and sequencing.
35. After the run, inspect each well individually in the PyroMark Q48 Autoprep software and flag any wells showing sequencing failures (e.g., low signal, background peaks, failed dispensation).

***Note:*** Exclude wells with sequencing failures from downstream analysis. Common causes include insufficient bead loading, degraded PCR product, or primer artifacts, see troubleshooting problem 4.

36. Export methylation data (percentage methylation per CpG) and normalize to the standard curve prepared from the methylated control DNA pools through linear regression (refer to Preparation 4).

**CRITICAL:** Normalize all mouse methylation data to the assay-specific standard curve within each run to account for non-linearities and run-to-run variation.

### *In vitro* methylation of pCpGL-CMV vector

**Timing: ∼5 h (hands-on: ∼30 min)**

In vitro methylation of the insert-containing pCpGL-CMV vector with M.SssI methylates all CpG sites in the insert, enabling functional assessment of the effect of CpG methylation on enhancer-driven luciferase expression.

37. Dilute SAM stock (32 mM) 1:20 in nuclease-free water to prepare a 1.6 mM working solution (1 μL SAM + 19 μL nuclease-free water). Prepare fresh on the day of use.
38. Set up methylation and mock reactions, materials and equipment, targeting 750–1000 ng plasmid per 10–15 μL reaction.
39. Incubate at 37°C for 4 h.
40. Heat-inactivate M.SssI at 65°C for 20 min.
41. Purify the methylated and mock-methylated vectors by drop dialysis on a 0.1 μm nitrocellulose membrane floating on Milli-Q water for ≥5 min.
42. Recover DNA droplet.
43. Quantify, dilute to 50 ng/μL in nuclease-free water.

**Pause point:** Methylated DNA can be stored at −20°C.

**CRITICAL:** Prepare methylated and mock-methylated vectors from the same DNA preparation in parallel to minimize batch-to-batch variability. The mock-methylated control is essential for calculating relative luciferase activity.

### Transfection of HEK293T cells and luciferase measurement

**Timing: 3–4 days**

HEK293T cells are co-transfected with the firefly luciferase reporter (methylated or mock) and Renilla-SV40 as a transfection efficiency control. Luciferase signals are measured 24 h after transfection using the Dual-Glo system. Transfection time can be extended by additional 24 h to a total of 48h of transfection.

44. Day 1: Cell Seeding
  a. Seed HEK293T cells in 96-well plates (if using white 96-well plates here, no transfer of the lysate during luciferase measurement is necessary) at 1 × 10^4^ cells per well in 100 μL antibiotic-free HEK293T growth medium per well.
  b. Incubate at 37°C, 5% CO_2_ for 16 h. Cells should be 80–90% confluent at the time of transfection (Day 2).

***Note:*** Antibiotic-free medium is required during transfection to avoid cytotoxicity from the interaction of Lipofectamine 2000 with antibiotics.

45. Day 2: Transfection
  a. Prepare Lipofectamine 2000–Opti-MEM mix: dilute 0.2 μL Lipofectamine 2000 in 4.8 μL Opti-MEM per well (see materials and equipment; scale up for required well number plus excess).
  b. Mix gently and incubate at 22°C ± 1°C for 5 min.
  c. Meanwhile prepare DNA–Opti-MEM mix per condition in PCR strip tubes (do not pre-mix all conditions in one tube):
    i. Add 2 μL plasmid (methylated or mock, 50 ng/μL) + 1 μL Renilla-SV40 (1 ng/μL) + 2 μL Opti-MEM per well (scale for number of reactions + excess).
  d. Combine 20 μL Lipofectamine–Opti-MEM (step 45a scaled) with 20 μL DNA–Opti-MEM (step 45c scaled) in the same PCR strip tube. Mix, briefly centrifuge. Incubate at 22 ± 1°C for 10–20 min.
  e. Using a multichannel pipette, dispense 10 μL of the transfection mix into each well of the 96-well plate (cells already contain 100 μL medium). Mix gently.
  f. Incubate at 37°C, 5% CO_2_ for 24 h.

**CRITICAL:** Each condition (methylated and mock for each insert) should be plated in technical triplicates. Multiple independent transfections on different days constitute biological replicates. Include appropriate transfection controls including background (transfection mix without any oligonucleotides) and empty pCpGL-CMV vector (accounting for background luciferase activity and additionally displaying the effects of the cloned enhancer).

46. Day 3: Luciferase Measurement
  a. Prepare Dual-Glo Luciferase Assay system reagents according to the manufacturer’s instructions.
  b. Add an equal to the medium amount (here 100 μL) of the Luciferase Assay reagent to the well.
  c. Lyse for 10 min at 22 ± 1°C. Shield plate from light.
  d. If a white plate was not used, transfer 180 μL of the lysate to a white plate appropriate for luminescence measurements.
    **CRITICAL:** Avoid bubbles when pipetting. Remove bubbles if visible
  e. Measure luminescence in a plate luminometer appropriate for 96-well formats.
  f. Add an equal to the medium amount (here 100 μL) or if 180 μL of the lysate were transferred add 90 μL of freshly prepared Stop & Glo Reagent.
  g. Incubate for 10 min at 22 ± 1°C. Shield plate from light.
  h. Read luminescence in a plate luminometer appropriate for 96-well formats.
  i. Export raw Firefly (FF) and Renilla (Ren) values.
  j. Subtract background from both FF and Ren values.
  k. Normalize each background subtracted FF signal to its corresponding Ren signal: normalized FF = FF/Ren.
  l. Take the average over the technical triplicates of mock and methylated value group. Adjust the averages of the normalized FF(mock or methylated) to the normalized FF(mock) within the same biological replicate.
  **CRITICAL:** The 1:1 ratio of Firefly to Renilla plasmid (100 ng : 1 ng) ensures robust Renilla signal for normalization without suppressing the experimental signal. Include appropriate transfection controls including background (transfection mix without any oligonucleotides) and empty pCpGL-CMV vector (accounting for background luciferase activity and additionally displaying the effects of the cloned enhancer).
  ***Alternatives:*** Instead of the Dual-Glo Luciferase Assay System, the Dual-Luciferase Reporter Assay System can be used.

## Expected outcomes

Following dCas9-KRAB transfection targeting RS1 with the specific sgRNA, a functional enhancer should produce a significant reduction in *Lep* mRNA expression compared to cells transfected with the scramble sgRNA control, indicating that RS1 actively contributes to *Lep* transcriptional regulation. Transfection efficiency in differentiated 3T3-L1 adipocytes is typically lower (30–70 %) than in proliferating cells, however some variability between biological replicates is expected. If transfection efficiency is too low, gene expression measurements from untransfected cells can obscure effects of successful transfection. *Hprt* Ct values should remain stable across conditions, confirming successful normalization. At least three independent biological replicates are recommended for statistical analysis.

Following bisulfite conversion and pyrosequencing, the PyroMark Q48 Autoprep software reports methylation levels at each CpG as a percentage based on T/C incorporation ratios. At the mouse *Lep* RS1 site in epididymal white adipose tissue, methylation levels typically range between 75–100% depending on the metabolic state and cohort. After normalization to the standard curve, data from multiple experimental runs and cohorts can be directly compared. An example pyrogram trace can be found in Figure 3.

For the luciferase assay, a methylation-sensitive enhancer should display significantly altered Firefly luciferase activity when the insert is M.SssI-methylated compared to the mock-methylated control (adjusted signal ratio <1). An enhancer that is insensitive to CpG methylation will show a ratio near 1. The empty pCpGL-CMV vector serves as a negative baseline and should produce minimal luciferase signal.

Representative expected outcomes, including reduction results and luciferase graphs can be found in the associated primary research publication.^1^

## Quantification and statistical analysis

All cell-culture experiments within the step-by-step method details should be performed at least as biological triplicates and technical duplicates; for the luciferase assay, we recommend technical triplicates as specified under Major step Transfection of HEK293T Cells and Luciferase Measurement. Specific number of mice included in the study can be found in the original research publication and is highly dependent on the expected power. However, for the aforementioned study, adipose tissue from 8 mice per group were used.

Pyrosequencing data are exported from the PyroMark Q48 Autoprep software as percentage methylation per CpG position per sample. Each sequenced well is individually quality checked before export. Raw percentages are entered into Microsoft Excel and normalized using linear regression or interpolation from the standard curve (prepared from 0%, 25%, 50%, 75%, and 100% methylated control DNA, see Preparation 2). Normalized methylation values from the same biological cohort are then analyzed statistically using an appropriate test for the experimental design (e.g., *t* test or Mann-Whitney U test for two groups; ANOVA with post hoc tests for multiple groups). Statistical significance is typically set at p < 0.05.

For luciferase data, the adjusted signal ratios to the average mock signal are calculated per biological replicate as described in the step-by-step method details under 46l. Values are presented as mean ± SD across biological replicates and tested for significant deviation from 1 (indicating methylation sensitivity) using a one-sample *t* test or Wilcoxon signed-rank test. Direct comparison between conditions uses paired tests where applicable.

## Limitations

The dCas9-KRAB experiment relies on successful transfection of fully differentiated 3T3-L1 adipocytes, which are post-mitotic and difficult to transfect. Transfection efficiency is typically low (30–70 %) compared to proliferating cell lines, which may dilute the observed effect on *Lep* expression. Additionally, dCas9-KRAB silences the targeted region through chromatin compaction rather than DNA methylation and therefore does not directly recapitulate the methylation-dependent mechanism assessed by pyrosequencing and the luciferase assay. Results from this approach should be interpreted as evidence for enhancer functionality in a genomic context, complementary to but distinct from the methylation-sensitivity data.

The bisulfite pyrosequencing protocol requires at least 60 mg of adipose tissue, which may not be available from all sample types, particularly from small or biopsy-limited sources. While the protocol includes provisions for reduced input with Carrier-RNA supplementation, very low DNA inputs (<50 ng) can result in variable bisulfite conversion efficiency and weaker PCR yields. Bisulfite conversion itself does not distinguish between 5-methylcytosine (5mC) and 5-hydroxymethylcytosine (5hmC); if 5hmC is expected to be present at the loci of interest, additional oxidative bisulfite sequencing (oxBS-seq) steps would be required.

The luciferase reporter assay evaluates the isolated RS1 insert in an artificial CpG-free plasmid backbone and overexpression context in HEK293T cells, which may not fully recapitulate the endogenous chromatin context or enhancer-specific proteins of adipose tissue. The assay detects the functional impact of global CpG methylation of the insert; it does not identify which specific CpG sites within the insert are responsible for the observed effect. M.SssI methylates all CpGs within the insert indiscriminately.

## Troubleshooting

### Problem 1: No or insufficient reduction in *Lep* expression after dCas9-KRAB transfection

Related to dCas9-KRAB transfection step (Major Step 1, 2).

### Potential solutions

• Verify transfection efficiency by including a GFP-expressing control plasmid in a parallel well and estimate the percentage of GFP-positive cells by fluorescence microscopy.
• Check differentiation efficiency before transfection; poorly differentiated cultures will show low *Lep* baseline expression, making further suppression difficult to detect.
• Optimize the transfection-reagent-to-DNA ratio for your specific cell passage number, as transfection efficiency in HEK and 3T3-L1 cells declines with increasing passage.
• Confirm that the scramble sgRNA control shows no reduction in *Lep* expression; if it does, the effect is non-specific and transfection conditions should be re-optimized. If the reduction persists, ordering a new scramble sgRNA might be necessary as off-target effects are likely.

### Problem 2: No or weak PCR product after bisPCR

Related to bisPCR step (Major Step 3–6).

### Potential solution

• Check bisulfite conversion efficiency by sequencing a non-CpG cytosine in the amplified region, this should appear as thymine after successful conversion.
• Increase the number of PCR cycles by 5 (up to a maximum of 45).
• Re-optimize the annealing temperature: test 2–3°C lower or higher in 1°C increments.
• Check DNA quality on the Qubit; debris or degraded DNA may be present; reisolate from stored tissue if possible.
• Ensure primers are stored correctly (−20°C, avoid freeze–thaw) and that biotin-labeled primer has not lost activity.

### Problem 3: Multiple or non-specific bands on agarose gel

Related to bisPCR step (Major Step 6).

### Potential solution

• Increase annealing temperature by 1–2°C increments.
• Reduce DNA input to the lower end of the range (10 ng per reaction).
• Add or increase Q-Solution in the master mix (see bisPCR Protocol, Q-Solution variant) to improve specificity.
• Check for homopolymer runs in the bisulfite-converted amplicon sequence: bisulfite conversion converts unmethylated cytosines to thymines, which can create extended thymine homopolymer stretches that reduce primer specificity and promote polymerase slippage. If homopolymers are identified, redesign primers to avoid these regions or shift the amplicon boundaries.
• Review primer length: bisulfite PCR primers (28–35 nt) are typically longer than standard PCR primers to compensate for the reduced sequence complexity of bisulfite-converted DNA. Primers that are too short may anneal non-specifically. If non-specific bands persist, redesign primers with increased length and verify predicted melting temperature using an in-silico bisulfite conversion tool prior to ordering.

### Problem 4: Pyrosequencing failure or low signal-to-noise ratio

Related to pyrosequencing step (Major Step 7).

### Potential solution

• Confirm that the PCR product loaded per well is within the acceptable range (10 μL of a strong, single-band product).
• Check that the biotinylated primer in the PCR is the correct one (the strand that becomes single-stranded during pyrosequencing capture).
• Re-validate the assay for primer interactions: if the sequencing primer + biotinylated primer combination produces signal, the assay should be redesigned.
• Ensure streptavidin-coated magnetic beads have not expired and were stored correctly.

### Problem 5: Low or absent luciferase signal after transfection

Related to transfection step (Major Step 9).

### Potential solution

• Verify cell confluency at transfection. Cells should be 80–90% confluent; over-confluent cells transfect poorly; under-confluent cells show toxicity.
• Confirm that medium used during transfection was antibiotic-free.
• Re-check the Lipofectamine 2000 expiry date and volume; too little reduces transfection efficiency, too much increases toxicity. Optimize Lipofectamine concentration with a test plate if transfecting a new cell batch.
• Verify that the Renilla-SV40 signal is detectable; if both signals are absent, the transfection itself has failed. Renilla-SV40 plasmid amount for transfection can be increased if Renilla signal is too weak.

### Problem 6: No difference between methylated and mock-methylated conditions in luciferase assay

Related to in vitro methylation and transfection steps (Major Steps 8–9).

### Potential solution

• Verify methylation efficiency: bisulfite-convert a small aliquot of both the methylated and mock-methylated vectors and pyrosequence with a CpG assay overlapping the insert to confirm >95% methylation in the M.SssI-treated vector.
• Confirm that the correct insert is cloned in the correct orientation by sequencing.

## Resource availability

### Lead contact

Further information and requests for resources and reagents should be directed to and will be fulfilled by the lead contact, Henriette Kirchner (henriette.kirchner@uni-luebeck.de).

### Technical contact

Technical questions on executing this protocol should be directed to and will be answered by the technical contact, Natalie Taege (n.taege@uni-luebeck.de).

### Materials availability

Plasmids and oligonucleotides generated in this study are listed in the key resources table. The pCpGL-CMV vector is available from Prof. Rehli’s laboratory and can be ordered through Addgene. Newly designed primers for the RS1 assays and sgRNAs used are provided in the key resources table.

### Data and code availability

The published article includes all datasets generated or analyzed during this study. Original source data for figures are available upon reasonable request from the lead contact.

## Acknowledgments

This work was supported by research funding from the 10.13039/501100001659German Research Foundation DFG (GRK 1957: Adipocyte-Brain Crosstalk).

## Author contributions

N.T., conceptualization, formal analysis, investigation, methodology, and writing – original draft; S.C.S., conceptualization, investigation, and writing – review and editing; H.K., conceptualization, writing – review and editing, supervision, project administration, and funding acquisition.

## Declaration of interests

The authors declare no competing interests.

## Declaration of generative AI and AI-assisted technologies in the manuscript preparation process

During the preparation of this work, the authors used Claude (Anthropic) to synthesize an initial draft from existing experimental protocols and methodological frameworks to enhance clarity and phrasing, and individual icons from the graphical abstract were generated. The AI-assisted output was subsequently reviewed, revised, and expanded by the authors to ensure scientific accuracy and completeness. The authors take full responsibility for the content of the published article.
